# Diversiform Etiologies for Post-stroke Depression

**DOI:** 10.3389/fpsyt.2018.00761

**Published:** 2019-01-23

**Authors:** Zan Wang, Yanmin Shi, Fangfang Liu, Nan Jia, Junya Gao, Xiaomin Pang, Fang Deng

**Affiliations:** Department of Neurology and Neuroscience Center, The First Bethune Hospital of Jilin University, Changchun, China

**Keywords:** post-stroke depression, depression, stroke, biological mechanism, social psychological mechanisms, default mood network

## Abstract

After the onset of stroke, many patients suffer from emotional behavior changes. Approximately, one-third of stroke survivors are affected by post-stroke depression (PSD), making it a serious social and public health problem. Post-stroke depression (PSD) has an important impact on the course, recovery, and prognosis of stroke. The pathogenesis of PSD is very complex, involving many factors such as biological mechanism and social psychological mechanisms. This article provides a brief review of the hot issues related to etiologies of PSD.

## Introduction

Post-stroke depression (PSD) refers to persistent depression after a stroke. Expressed as loss of interest, decreased energy, decreased appetite, sleep disorders, low self-evaluation, self-blame, and even repeated self-injury, suicidal thoughts or behaviors. It is the most common emotional disorder after stroke. As early as 1977, Folstein et al. reported PSD for the first time, and its incidence rate was as high as 45% (1). Patients with major depression after stroke account for 10–25% of stroke patients, and those with mild depression account for 10–40% of stroke patients. Symptoms were most common in the third month after stroke, and the prevalence did not decrease in the following year (2). The clinical manifestations of patients with post-stroke depression (PSD) are more complicated. Clinically, the patient's performance is often divided into core symptoms and non-core symptoms. The main symptoms are: (1) most of the time patients feel unhappy, even painful; (2) lose interest and pleasure, and cannot get happiness from the things they usually love; (3) energy decline, easy to feel tired, even lose the belief of living, suicidal tendencies. Non-core symptoms are mainly: (1) weight loss, difficulty sleeping, insomnia and dreams, unexplained loss of appetite, pain, general malaise; (2) nervousness, anxiety; (3) self-evaluation decline, self-blame, worthless, hesitant, attention decreased, etc. The diagnosis of typical cases of PSD is not difficult. Patients with a history of stroke, low mood, lack of interest or loss of fun, plus some psychological, or physical symptoms can make a diagnosis. A considerable number of patients do not show obvious sadness and despair, but mainly a variety of physical symptoms, such as fatigue, anxiety, tension headache, loss of appetite, sleep disorders. Post-stroke depression affects the patient's cognitive function and quality of life, increases the patient's mortality and self-killing rate, and imposes a heavy burden on society and the family. However, there are still many ambiguities about the risk factors, etiologies of PSD. Therefore, early accurate etiologies of PSD is very important and should be taken seriously by clinicians. The research progress in the incidence, etiologies is summarized as follows.

## Incidence and Prevalence of PSD

There are significant differences in the incidence of PSD, due to differences in study selection, time to assessment after stroke, assessment methods and diagnostic criteria (3). A systematic review of 14 studies involving the prevalence of PSD found that the peak of depression was 3–6 months after stroke, and the prevalence was 9–34%. The prevalence of depression remained at a high level until 1–3 years after stroke; the prevalence of mild depression after stroke was ~8–22% (4). Hackett analyzed 51 studies: Using the Hamilton Depression Scale (HDRS), the lowest PSD rate was 26%. The highest incidence of PSD was 41% with the Montgomery-Asberg Depression Rating Scale and the Zung Depression Scale (5). Schöttke believes that the incidence of PSD was 31.1%, post-stroke anxiety prevalence was 20.4% (6). Chemerinski et al. analyzed 24 studies and classified patients with strokes from different sources. The results showed that the incidence of major depression in acute hospitalized stroke patients was 22%, mild depression was 17%; Out-patient stroke with severe depression was 23%, mild depression was 35%; community patients had severe depression of 13% and mild depression was 10% (7).

## Etiologies of PSD

The pathogenesis of PSD is complex, involving many factors such as biological mechanism and social psychological mechanisms.

### Biological Mechanism

#### Monoamine Neurotransmitter Change

Numerous studies on depression have confirmed that noradrenergic and serotonergic neurons involved in emotional regulation in the brain are located in the brainstem. Its axons pass through the hypothalamus, basal ganglia, corpus callosum, and radial crown, and finally reach the frontal cortex. 5-HT and NE are both monoamine neurotransmitters, mainly involved in depression, anxiety, self-injury suicidal behavior, and sleep disorders. When stroke destroys the above related structures, it can cause a decrease in NE and 5-HT levels, and patients are more prone to depression (4). Some scholars have found that the concentration of serotonin metabolites in cerebrospinal fluid of patients with PSD is reduced (8). Combined with clinical application of antidepressants such as selective serotonin reuptake inhibitors (SSRIs), it is effective in the treatment of PSD. It was further confirmed that the occurrence of PSD is associated with a decrease in monoamine neurotransmitters (9).

Studies have found that the occurrence of PSD is related to neurotransmitters such as glutamate (Glu) and gamma-aminobutyric acid (GABA). Hypoxia-induced hypoxia causes a decrease in ATP and changes in membrane permeability leading to K+ efflux and Ca2+ influx, leading to an increase in excitatory amino acids such as glutamate, while re-uptake is blocked, excitatory amino acids accumulate outside the cell, leading to post-synaptic Excessive excitation, degeneration, and necrosis of neurons. Increased Glu leads to post-synaptic neurons, excessive excitability, ulceration, and necrosis. Patients with PSD are often accompanied by changes in the level of glutamate in the frontal lobe. PSD patients have a significantly elevated glutamate/creatinine ratio in the frontal lobes and anterior cingulate gyrus. This change is associated with the Hamilton Depression Scale (HDRS) is associated with patients with high scores (10). Wang et al. used MRI spectroscopy studies to show that patients with PSD have higher glutamate levels than stroke patients without PSD (11). The mechanism of PSD is related to the imbalance of GABA expression, and a decrease in GABA can lead to a low level of NE (12).

#### Inflammation Mechanism

Inflammation refers to the defense response of living tissue to the stimulation of biological, physical, chemical, and other damage factors. Various inflammatory factors refer to cytokines involved in the inflammatory response, and are hydrophilic specific polypeptides or small molecular proteins secreted by activated immune cells. When inflammation occurs in the body, inflammatory factors inactivate the phosphorylation of the inhibitor of nuclear factor kappa B (IκB) resulting in a decrease in the inhibition of nuclear factor kappa B (NF-κB) by IκB, which causes NF-κB to enter the nucleus and bind to specific NF-κB. Inducing the transcription of related genes and promoting the expression of genes, causing an increase in anxiety and other depressive behaviors. IL-1, IL-2, IL-6, and TNF-α are mainly produced by mononuclear macrophages, which are involved in the inflammatory response and promote the immune response. Both IL-10 and IL-13 are important inhibitory cytokines, mainly produced by Th2 cells, which inhibit the production of pro-inflammatory cytokines, suppress immune responses, and protect nerves. Inflammation plays a protective role in maintaining the homeostasis of the body. However, if it is overreacted, it can damage normal tissues and organs. Studies have found that inflammation under certain conditions can damage the internal balance of the body, causing metabolic disorders, leading to abnormal secretion of neurotransmitters, resulting in depression (13). Spalletta et al. believe that the occurrence of PSD may be related to immune activation, leading to increased secretion of cytokines, and proposed a “cytokine hypothesis.” After stroke, astrocytes and microglia in the central nervous system produce cytokines and their receptors, including IL-1, IL-6, TNF-α, and IFN-γ. The phenomenon of immune activation and increased cytokines (14).

Studies have suggested that inflammatory factors may cause depression through neurodegeneration, decreased regeneration, decreased ω-3 fatty acids, decreased levels of tryptophan, and elevated levels of metabolites. Inflammatory factors interact with each other to form a network system that regulates immune responses (15, 16). Inflammatory factors can increase the activity of indoleamine-2,3-dioxygenase (IDO), increase the metabolism of tryptophan, and increase the concentration of quinolinic acid and kynurenine. The level of serotonin precursors synthesized by tryptophan is reduced, causing a decrease in serotonin concentration and accelerating depressive symptoms in patients (17, 18).

Yang et al. studies found that IL-18, IL-1, IL-6 play an important role in the occurrence, development and prognosis of PSD (19). Kim et al. performed polymorphisms of pro-inflammatory cytokine genes such as IL-1β, IL-6, IL-8, TNF-α, and polymorphisms of anti-inflammatory cytokines such as IL-4 and IL-10 in patients with PSD. The results showed that the IL-10-1082A/A genotype was closely related to PSD, and the IL-4 + 33C/C genotype was only associated with heavy PSD (20). After a year of follow-up of PSD patients, Su et al. found that IL-10 levels in the depression group were significantly lower than those in the non-depressed group, and IL-10 levels were negatively correlated with depression. IL-10 may have antidepressant effects (21). Spalletta et al. believe that stroke promotes the release of inflammatory factors such as C-reactive protein (CRP), interleukin-1(IL-1), tumor necrosis factor-α (TNF-α), IL-6. These factors stimulate and produce a toxic effect on the monoamine neurotransmitter system. As a result, its function declines, causing depression to occur (22). Increased expression of TNF-α, IL-1β, and cortisol releasing factor was found in the hippocampus of PSD rats, and interaction between cortisol releasing factor and TNF-α signaling pathway was found in PSD (23). Studies by Reichenberg et al. have shown that induction of TNF-α production by experimental stimulation can induce depression and cognitive function changes in humans (24).

#### Hypothalamus-Pituitary-Adrenal Axis and Hypothalamus-Pituitary-Thyroid Axis

Studies have shown that inflammation can affect the function of the hypothalamic-pituitary-adrenal axis (HPA), promote the metabolism of monoamine transmitters, and induce depression (14). Pro-inflammatory factors activate HPA and promote excessive secretion of cortisol, and excess cortisol damages nerve cells through cytotoxicity. Abnormal activity of the HPA and hypothalamus-pituitary-thyroid axis (PHT), leading to elevated plasma cortisol. Elevated plasma cortisol induces the production of tryptophan pyrrolase and aminotransferase in the liver, which both degrade blood tryptophan (5-HT precursor) and tyrosine (NE precursor), resulting in 5-HT and NE synthesis are reduced, promoting, or aggravating the occurrence and development of PSD (25–27). Persistent and excessive secretion of cortisol can inhibit hippocampal neuronal regeneration and reduce neural plasticity in the pre-frontal cortex, leading to PSD.

#### Glial Cells

##### Astrocytes

Glial cells are widely distributed in the central and peripheral nervous systems and are mainly divided into three types: astrocytes, oligodendrocytes, and microglia. Astrocytes are the most important glial cells, which secrete a variety of neurotrophic factors, and play an important role in energy metabolism regulation, neurotrophic factor release, neuronal synaptic remodeling and nerve formation. A large body of evidence indicates that astrocyte dysfunction is an important factor in the onset of depression. Studies have reported that astrocyte hyperplasia is a characteristic response to central nervous system inflammation or injury. Astrocytes and microglia work together to regulate the release of pro-inflammatory cytokines and anti-inflammatory cytokines, maintaining the normal physiological functions of the brain. Astrocytes can release a variety of neurotrophic factors, such as nerve growth factor, brain-derived neurotrophic factor (BDNF), glial-derived neurotrophic factor (GDNF), fibroblast growth factor 2 (FGF 2) etc. Such neurotrophic factors regulate nerve function, promote nerve growth, and increase synaptic plasticity and delivery efficiency (28). Neurotrophic factor is a molecule that promotes the development and survival of neurons. It can prevent the pathological changes of ischemic brain injury, and can reduce the apoptosis of neurons and effectively improve the neurological function of patients after stroke. Previous experimental studies have found that neurotrophic factors are closely related to the condition and prognosis of PSD (29). A number of studies have found that levels of neurotrophic factors are significantly reduced in patients with PSD (30). A meta-analysis study showed a decrease in the expression of GDNF in the brain of patients with depression. Studies have shown that the reduction of GDNF and BDNF levels in the brain of patients with depression is associated with decreased hippocampal nerve regeneration. Antidepressants can increase the production of BDNF and GDNF in rat hippocampus (31–33). Selective serotonin reuptake inhibitors (SSRIs) significantly increased the expression of BDNF mRNA in astrocytes, suggesting that antidepressants can exert antidepressant effects by increasing astrocyte BDNF synthesis. Fluoxetine can increase the synthesis of GDNF and BDNF in astrocytes; amitriptyline can promote the synthesis and release of astrocytes FGF-2, BDNF, and GDNF (34, 35). Other studies have shown that astrocytes can synthesize antibodies and anti-inflammatory factors and inhibit the synthesis of pro-inflammatory factors. Astrocyte dysfunction can aggravate the inflammatory response and aggravate central nervous system damage (36).

##### Microglia

Microglia are immune cells of the central nervous system. When tissue damage or brain infection occurs, microglia are first activated to perform functions such as antigen recognition, phagocytosis, and antigen presentation (37). When activated, microglia can produce a large number of pro-inflammatory factors, causing degeneration and necrosis of neurons. Under normal circumstances, microglia are in a resting state, receive synaptic signals by sensing changes in the extracellular environment, thereby participating in intersynaptic interactions; and can also express neurotransmitters such as dopamine and serotonin. When the extracellular environment changes, microglia can be activated and undergo morphological changes, releasing inflammatory factors (38). Activated microglia are divided into two polarization states, M1 and M2. Polarized M1 microglia produce pro-inflammatory cytokines and neurotoxicity participate in the development of neural network dysfunction and promote inflammation. Polarized M2 microglia secrete anti-inflammatory mediators and neurotrophic factors involved in restoring homeostasis (39). Previous studies have shown that autopsy after suicide in patients with major depression found activation of microglia in the pre-frontal cortex and lateral anterior cingulate gyrus. Moreover, activation of microglia in the anterior cingulate cortex, hippocampus, and thalamus is associated with suicide caused by depression (40). The cause of depression is related to the secretion of pro-inflammatory factors by M1 microglia. A variety of antidepressants have anti-inflammatory effects and can reverse the M1 type polarization of microglia. Fluoxetine and citalopram, widely used clinically, regulate the immune system by inhibiting M1 polarization and improving M2 polarization of microglia, mediating the therapeutic effects of drugs (41).

#### Vitamin D

Vitamin D is a neurosteroid hormone, 25-hydroxyvitamin D is its main form in the blood circulation (42). Vitamin D is derived from food, especially from fish oil, which is synthesized on the skin and is affected by light. Vitamin D receptor (VDR) is located in an important area of the brain associated with depression and emotional behavior, such as cingulate gyrus, hippocampus, thalamus, hypothalamus, and substantia nigra (43). VDR is also present in immune cells and has an immunomodulatory effect (44). Vitamin D can regulate neurotransmitters such as serotonin in the brain through tryptophan-hydroxylase 2, vitamin D deficiency may lead to central morphological changes and decreased synthesis of neurotransmitters such as norepinephrine and dopamine (45). Studies by Puchacz et al. showed that vitamin D is involved in the regulation of the expression of the tyrosine hydroxylase gene, which catalyzes the production of levodopa by tyrosine in dopamine biosynthesis (46). Studies have found that vitamin D levels are negatively correlated with inflammatory markers, and the relationship between depression and inflammatory response can be regulated by the immune system (47). In the central nervous system, vitamin D acts as a neuroprotective factor through its antioxidant activity to increase the efficiency of neuronal projection and regulate the synthesis of neurotransmitters. A meta-analysis found that low vitamin D levels are associated with depression levels and are the biological basis for depression susceptibility (48). Studies have shown that supplemental reduced vitamin D levels contribute to the improvement of depressive symptoms in patients with depression, but different studies have also been reported (49). Han et al. also believe that vitamin D levels are positively correlated with PSD within 24 h after stroke onset (50). A prospective randomized controlled clinical trial by Shaffer et al. found that vitamin D supplementation helps prevent PSD. High serum vitamin D levels protect patients from PSD, and recent randomized controlled trials have shown that vitamin D supplementation can improve depressive symptoms in patients (51, 52).

#### Homocysteine

Some studies have concluded that high homocysteine levels are significantly associated with PSD. Stroke patients with high levels of homocysteine are relatively more prone to PSD (53). High homocysteine (Hhcy) has a direct toxic effect on blood vessels, causing further damage to the cerebral blood vessels, leading to the occurrence of depression (54). Hhcy affects the production and metabolism of monoamine neurotransmitters such as DA, 5-HT, NE, etc. These neurotransmitters play an important role in the pathogenesis of depression (55). Liu et al. studied 18 patients with ischemic stroke and selected three core regions of DMN (left parietal cortex, pre-frontal cortex, posterior cingulate ganglion/wedge anterior cortex). Then, the difference between the patient and the normal person ReHo was compared, and the ReHo of the posterior cingulate cortex of all stroke patients was found to be reduced. The functional connectivity (FC) analysis was performed using the posterior cingulate cortex for the region of interest, and the FC values of posterior cingulate cortex and anterior cingulate were found to be reduced (56).

#### Neural Network Dysfunction

Resting-state functional magnetic resonance imaging (rs-fMRI) indirectly reflects the functions of brain local and neural networks through signal changes, which have the advantages of non-invasiveness and reproducibility. Since the early 1990s, it has had an important impact on the development of neuroscience and psychology. In recent years, it has also begun to be used in the research of diseases such as PSD, and has gradually become one of the important means for studying the physiological and pathological activities of brain function. At present, the commonly used analytical methods for rs-fMRI are as follows: Regional homogeneity (ReHo), Amplitude of low-frequency fluctuation (ALFF), Functional connectivity (FC) (57). The most widely studied is the default mood network (DMN), which mainly involves the medial pre-frontal cortex, posterior cingulate gyrus/pre-wedge lobes, bilateral apical lobes (including angular gyrus), bilateral lateral temporal lobe, hippocampus, etc. Studies have shown that the default network is closely related to the monitoring of internal and external environments, the processing of emotions, introspection, the maintenance of thinking cognition, and the extraction of thought memories. A large number of studies have shown that there is an abnormality in the brain network under the resting state of depression, and DMN is an important neuropathological mechanism of depression (58, 59). Zhang et al. performed FC analysis on patients with cerebral infarction, and found that compared with non-PSD and normal people, PSD patients had changes in the right frontal gyrus and the left gyrus and the anterior cingulate gyrus. In addition, the FC values of the left temporal and anterior cingulate gyrus were significantly associated with the severity of depression (60). Liu et al. (56) studied 18 patients with ischemic stroke, and selected 3 core regions of DMN (left parietal cortex, pre-frontal cortex, posterior cingulate gyrus/anterior cranial cortex), and then compared patients and normal Human differences. The ReHo of the posterior cingulate cortex was found to decrease in all stroke patients, and the posterior cingulate gyrus was used as a functional area for FC analysis, and the FC value of the posterior cingulate gyrus and the anterior cingulate gyrus was found to be reduced (56). Zhang et al. studied 26 patients with PSD, and the results showed that the default emotional network, cognitive control network, and emotional network FC of PSD patients changed. The left lower parietal lobe, the left eyelid portion of the inferior temporal gyrus and the left anterior gyrus were significantly associated with the Hamilton Depression Rating Scale for PSD patients. Changes in the three neural networks may be associated with the development of PSD in the subacute phase of stroke (61).

At present, there are many studies on the relationship between PSD and stroke sites, but the conclusions are not the same. Current brain imaging studies of depression have shown that subcortical white matter damage leads to a susceptibility to depression by destroying certain neural circuits associated with emotions. Depression brain function changes mainly in the pre-frontal cortex, anterior cingulate gyrus, amygdala, ventral striatum, hippocampus, insula, thalamus, and basal ganglia. The pre-frontal cortex is thought to play a key role in cognitive and emotional activities, and functional abnormalities in these brain regions may present with affective and cognitive impairments. Current studies have shown that left hemisphere stroke is more prone to depression than right hemisphere stroke. The lesions near the frontal pole have a specific correlation with the degree of PSD. The lesions associated with PSD were: frontal lobe, left basal ganglia, and temporal lobe, and the incidence of lesions near the extreme was high (62). Carson et al. found that the occurrence of PSD was not related to stroke lesions after evaluation of related studies (63). PSD has nothing to do with the stroke site. The reasons for the difference between the two may be different sample sizes, differences in diagnostic criteria, ethnic differences, geographical differences, and so on (64). Vataja et al. found that the lesions of acute stroke were located in the left hemisphere, especially in the left anterior hemisphere, and their chance of developing depression was higher than in the right hemisphere. It is believed that the damage of the globus pallidus and the volume of injury are related to the diagnosis of depression within 3 months after stroke (65). Angeleri et al. observed the observation of the 3 years after the stroke, 3 years or more after stroke, the incidence of depression is not related to the lesion in the left or right hemisphere (66). Shimoda et al. observed the relationship between lesions and PSD at different time points after stroke. In the acute phase of stroke, PSD was associated with left anterior hemisphere lesions; at 3–6 months, PSD was related to the distance from the lesion to the frontal pole and lesion volume; and 1–2 years later, PSD was related to the distance from the right hemisphere lesion to the occipital pole and lesion volume (67).

Cognitive dysfunction is one of the common complications in stroke patients. Most studies have concluded that depression has a significant relationship with cognitive dysfunction. However, the specific relationship between the two is still controversial. Many studies have identified the most relevant factors for PSD and cognitive impairment: low education, speech impairment, stroke severity, and previous diabetes history (68). Murata et al. found that cognitive function improved with the improvement of PSD symptoms (69).

#### Genetic Background

Regarding the gene hypothesis, there is clear evidence that the shortening of the promoter region associated with the serotonin gene is associated with severe PSD. Studies have shown that individuals and families with a history of depression may be one of the risk factors for major depression after stroke (70, 71). The expression of some genes is considered to be a risk factor for PSD. Brain-derived neurotrophic factor (BDNF) plays an important role in the pathophysiology of PSD. It has been suggested that the single nucleotide polymorphisms rs1778929 and rs1187323 in the tyrosine receptor kinase B (TrkB) gene of BDNF are significantly associated with PSD (72). Kim et al. evaluated 222 stroke patients and followed up for 1 year. The increase in methylation status of 5-HTTLPR (The serotonin transporter-linked polymorphic region, 5-HTTLPR) SS genotype was associated with PSD. Higher levels of BDNF gene methylation were associated with PSD occurring at follow-up (73). The serotonin transporter gene (SLC6A4) has also been shown to play an important role in the pathophysiology of PSD. Studies have found that 2 weeks after stroke, higher SLC6A4 promoter methylation status is independently associated with PSD and is more pronounced 1 year after stroke, and is significantly associated with worsening depressive symptoms within 1 year (74). Studies have shown that apolipoprotein E (APOE) polymorphism is associated with PSD, APOE rs429358 polymorphism increases the probability of PSD, APOE rs429358-C allele may be post-stroke nerve Functional recovery is harmful (75).

### Social Psychological Mechanisms

After cerebrovascular disease, most patients have different degrees of physical dysfunction, resulting in loss of work and life. The combined effects of family, society, and physiology lead to physiological and psychological imbalance in stroke patients. Psychosocial factors such as poor living ability, negative life events, family burden, social family support may all contribute to PSD. Acute stroke is a stressful event that increases the secretion of glucocorticoids, causing elevated blood glucose and abnormal neurotransmitters, leading to depression (76). The onset of PSD is not a single mechanism. Whyte et al. proposed that PSD, like other psychiatric diseases, is under the bio-psycho-social medical model, and that biological factors and psychological factors may contribute to the onset of PSD (4).

Studies have shown that the degree of education is negatively correlated with the occurrence of PSD, probably because patients with low levels of education have limited cognitive levels (77). Studies by Backhouse et al. showed that lower education levels were associated with an increased risk of PSD symptoms, but confidence intervals and heterogeneity were greater (78).

The relationship between age and PSD has been controversial. Previous studies have found that the younger patients with acute stroke, the higher the risk of PSD. This may be due to young people taking on greater family and social responsibility. After the stroke, the social roles and economic status of young patients are more prominent and the psychological acceptance is poor (79). Some studies have shown that age is positively correlated with the occurrence of depression. With the increase of age, the body's various functions are declining, frustration and attention to the body become more and more prominent. There are also many studies that do not have a clear correlation between age and PSD (80).

Studies have shown varying incidence rates for patients of different genders. Most studies have found that women are more likely to develop PSD earlier than men. This may be related to women's poor psychological quality, sensitivity, psychological and physiological imbalance. Another possible explanation is that women live longer than men, so women have an average age greater than men when they encounter a stroke (81). However, another study found that the prevalence of male PSD is higher than that of females. It may be that men have more family and social responsibility for men. Therefore, changes in work ability and social status caused by stroke will produce greater psychological stress in men (82). However, another part of the study concluded that there is no difference between the two (83).

In the study of pre-existing personality characteristics of patients with PSD, it is found that patients with neuroticism, introversion, emotional instability, and strong dependence are more likely to develop PSD (84). Pessimism, negative coping, introversion, acceptability, etc. are independent risk factors for PSD.

Regarding the marital status of patients, studies have shown that widowhood, divorce, and solitary living are closely related to depression. It may be related to the patient's loneliness, social isolation, long-term physical illness, and decreased stress ability.

The severity of stroke directly affects the quality of life of patients and has a great impact on the occurrence of PSD. Many studies have shown that the ability of daily living is related to the occurrence of PSD in the early stage of stroke. The more severe the neurological damage, the lower the ability of daily living, the greater the risk of PSD. There was a significant correlation between the activities of daily living (ADL) and the incidence of depression after stroke. Low ADL is an important factor leading to PSD. The severe physical dysfunction, low self-care ability and loss of working ability make patients have great psychological pressure (85).

Studies on chronic diseases and depression have shown that chronic diseases such as hypertension, diabetes, dyslipidemia, and respiratory diseases are also in the category of psychosomatic diseases. Due to the long course of chronic diseases and the difficulty of treatment, patients are often in a state of anxiety and depression. Most patients have low ability to recognize these mental illnesses. Current research shows that among vascular risk factors, only hypertension can predict PSD. Diabetes, hyperlipidemia, obesity, and smoking were not independent predictors of PSD (86).

Social support can be divided into two categories, one is objective, visible or practical support; the other is subjective and empirical emotional support. Good social support will force the patient's psychological endurance, indirectly promote the recovery of stroke patients and improve their quality of life, while stroke patients who lack social support are more likely to develop PSD (87). Kotila et al. found that patients who lived in community-active areas after stroke had fewer PSD than those without community activity, suggesting that appropriate rehabilitation activities can reduce PSD (88).

## Conclusion

PSD seriously affects the quality of life of patients, which is a burden on individuals, families, and society. It is necessary to explore the pathogenesis and related influencing factors, establish appropriate diagnostic criteria and scales, and determine the best preventive and therapeutic measures. The pathogenesis of PSD is extremely complex and may be the result of multiple factors and multiple pathways. There are still many uncertainties in the neurobiological mechanisms of PSD. It is believed that with the improvement of science and technology, people will gradually uncover the mystery of the biological mechanism of PSD and provide a more accurate theoretical basis for the diagnosis and treatment of PSD.

## Author Contributions

YS and ZW wrote the main manuscript text, contributed equally to this work and should be regarded as co-first author. FD contributed substantially to the conception and design of this work, drafting of the work, and revised it critically for important intellectual content. FL, NJ, JG, and XP collected the data etc.

### Conflict of Interest Statement

The authors declare that the research was conducted in the absence of any commercial or financial relationships that could be construed as a potential conflict of interest.
